# Multi-Fidelity Aerodynamic Data Fusion with a Deep Neural Network Modeling Method

**DOI:** 10.3390/e22091022

**Published:** 2020-09-12

**Authors:** Lei He, Weiqi Qian, Tun Zhao, Qing Wang

**Affiliations:** Computational Aerodynamics Research Institute, China Aerodynamics Research and Development Center, Mianyang 621000, China; leiluodelei@nudt.edu.cn (L.H.); qianweiqi@cardc.cn (W.Q.); wangqing_mail@163.com (Q.W.)

**Keywords:** aerodynamic data fusion, multi-fidelity data, machine learning, deep neural networks, variable complexity modeling, cokriging

## Abstract

To generate more high-quality aerodynamic data using the information provided by different fidelity data, where low-fidelity aerodynamic data provides the trend information and high-fidelity aerodynamic data provides value information, we applied a deep neural network (DNN) algorithm to fuse the information of multi-fidelity aerodynamic data. We discuss the relationships between the low-fidelity and high-fidelity data, and then we describe the proposed architecture for an aerodynamic data fusion model. The architecture consists of three fully-connected neural networks that are employed to approximate low-fidelity data, and the linear part and nonlinear part of correlation for the low- and high-fidelity data, respectively. To test the proposed multi-fidelity aerodynamic data fusion method, we calculated Euler and Navier–Stokes simulations for a typical airfoil at various Mach numbers and angles of attack to obtain the aerodynamic coefficients as low- and high-fidelity data. A fusion model of the longitudinal coefficients of lift $C_{L}$ and drag $C_{D}$ was constructed with the proposed method. For comparisons, variable complexity modeling and cokriging models were also built. The accuracy spread between the predicted value and true value was discussed for both the training and test data of the three different methods. We calculated the root mean square error and average relative deviation to demonstrate the performance of the three different methods. The fusion result of the proposed method was satisfactory on the test case, and showed a better performance compared with the other two traditional methods presented. The results provide evidence that the method proposed in this paper can be useful in dealing with the multi-fidelity aerodynamic data fusion problem.

## 1. Introduction

In general, aerodynamic data are the main source for engineers to obtain aerodynamic performance information of aircraft, and they are generated via three types of aerodynamic testing: flight testing, wind-tunnel testing, and computational simulations [1]. Different information is provided from different sources. Flight testing can obtain the most accurate and reliable information and is often used as a final assessment; however, the flight testing is expensive, and the test cycle is long. Therefore, during the engineering development phase, aerodynamic development and analysis rely on wind-tunnel testing. Wind-tunnel testing is an important means to simulate the performance of aircraft, including the prediction of aerodynamic force/heat inside the flight envelope and the establishment of an aerodynamic database, confirming the reliability of the numerical simulation results [2]. Although the test data accuracy is relatively accurate, wind-tunnel testing is not cheap to perform in terms of cost, time or resources.

Computational fluid dynamics (CFD) is also widely applied in the aerodynamic engineering field to simulate the performance of systems, saving the cost of expensive wind tunnel experiments. In recent years, benefiting from the development of computer technology, the ability of the CFD method to simulate complex physical problems has been rapidly and constantly improving. However, such computational experiments still consume time to produce a large amount of accurate aerodynamic data and often show discrepancies in the results compared with experiments, particularly with low-fidelity computational models. Therefore, the CFD method is commonly conducted at an initial stage of aerodynamic development and is often examined by wind-tunnel testing. Combining the information provided by different fidelity data sets is often necessary to successfully produce high-quality aerodynamic data [3].

The aerodynamic characteristics are closely related to the configuration and flow field. Compared with the conventional sensor measurement data, aerodynamic data has its own complexity and particularity. Modeling-based aerodynamic data fusion methods typically need to construct a mathematical model that can describe the aerodynamic characteristics that the data fusion operation is based on. Data modeling methods are often divided into two groups: conventional aerodynamic modeling methods with explicit physical meaning and surrogate-based aerodynamic modeling methods [4]. The aerodynamic data fusion steps based on conventional models with explicit physical meaning are: the analysis of the aircraft aerodynamic characteristics, the construction of aerodynamic model, and the parameter identification of models using data from different sources.

The advantages of this method are that the model can reflect the aerodynamic characteristics in the level of physical laws and the model has the anti-interference ability on the aerodynamic noise data. The deficiencies are that the modeler must have a deep understanding of the physical theory of aerodynamics and the modeling period is always long. Surrogate-based aerodynamic data fusion method require the construction of a surrogate model. The surrogate model is more sensitive to noise data, which causes its anti-interference ability to be inferior to the former. However, the modeling efficiency is higher and the modeling time is shorter. With the development of computer technology and wind tunnel test technology, the quality of the aerodynamic data are continually increasing, and the interference amplitude noise data are decreasing.

Surrogate models are often applied in the domain of engineering design and optimization of aerospace, which can statistically approximate the relationship between a set of design variables and their response, resulting in reducing the resources required for design, search, and optimization. The surrogate models commonly used include polynomial response surface (PRS), spatial correlation models (or “kriging”), multivariate adaptive regression splines (MARS), regression trees and boosting, radial basis function (RBF) networks, and least interpolating polynomials [5,6,7,8,9,10]. Surrogate modeling is widely used to describe the performance of a system, due to its simplicity and efficiency [11,12].

A surrogate model is an approximation of the relationship between the input variable and the response of a certain system [13], and they are often used in the initial development phases to reduce the resources required for design and optimization [14,15]. However, most surrogate models still have limitations in dealing with high-dimensional problems or large scale data [16].

Taking kriging as an example, the time-consumption will increase significantly with the increase of training samples. It is also difficult for ordinary computers to support the matrix operations required by the kriging model as the amount of data points increase to a large scale. There are many matrix operations that exist in the process of building a surrogate model with the kriging method, in particular the inverse operation of the covariance matrix. If the amount of data points used to build the kriging surrogate model is n, the size of the covariance matrix is n × n. In engineering practice, the amount of low-fidelity data points often reaches tens of thousands or even more; therefore, the covariance matrix involved in the model-building process would be even larger and the matrix operation would be more difficult. Over the past years, deep neural network models have become increasingly practical and important for the development of deep learning approaches. Deep neural networks have shown great successes in dealing with large-scale data. They can easily handle linear or nonlinear problems at both low- and high-dimensions [17].

In this paper, we applied the deep neural network algorithm in fusing aerodynamic data with different fidelity levels. The paper is organized as follows. In Section 2, we discuss the related work, in particular regarding variable complexity modeling (VCM) and the cokriging method used to handle the aerodynamic data fusion problem. In Section 3, we provide the details for the multilayer perceptron (MLP) and architecture of air aerodynamic data fusion-based deep neural networks along with the training processes. In Section 4, the aerodynamic force data fusion results of a typical airfoil sharp with different methods are discussed, and the accuracy is analyzed. Our concluding remarks are provided in Section 5.

## 2. Related Work

Various surrogate modeling methods have been studied to fuse the information provided by multi-fidelity aerodynamic data. Hutchinson [18,19] studied a variable-complexity strategy with a scaling factor of combining simple and detailed analysis methods for the design optimization of a high-speed civil transport (HSCT) wing. A. J. Keane et al. [20] used a fusion-based method with an experimental design to solve the transonic wing optimization problem. The key of the method was to build a response surface (RSM) model of the differences between the empirical and CFD data, which have different levels of fidelity. The fusion-based method was shown to be more accurate than the initial empirical model or a simple RSM using only data from CFD. Stephen J. Leary et al. [21] described several a priori knowledge-based approaches to multi-fidelity modeling problems, in particular, the knowledge-based artificial neural networks and a new knowledge-based kriging model. Approaches that used a low-fidelity model as the prior knowledge were more effective than RSM approaches built on expensive models alone. C.Y. Tang [22] applied a variable complexity modeling method with an increment function to merge various fidelity solutions into a single, coherent database. A crew transfer vehicle (CTV), which can provide an excellent test case for the generation of aerodynamic data for its flight envelope and contains subsonic, transonic, and supersonic flows, was selected to evaluate the method. Jun Zheng et al. [5] constructed a hybrid variable-fidelity global approximation model to fuse data, where RBF was used to approximate the low-fidelity data and kriging was used to build a correction model. Maxim Tyan et al. [23] also studied the data fusion approach to construct aerodynamic tables for flight simulation using data obtained from various sources, and an additive scaling function was created to correct low-fidelity functions to match the high-fidelity data in this approach. Renganathan et al. [24] proposed a Bayesian framework to fuse two aerodynamic data sets originating from differing fidelity physical or computer experiments that could be corrupted by noise, bias, and incompleteness. M. Ghoreyshi et al. [25] described a framework based on sampling and data fusion technology to generate aerodynamic tables for flight simulation. The cokriging method was used in this study to build a data fusion model for combining samples from different fidelity sources that were expensive and cheap to evaluate. Y. Kuya et al. [26] constructed a multi-fidelity surrogate model of an inverted wing with vortex generators (VGs) in the ground effect based on cokriging regression to combine wind-tunnel experimental data and Reynolds Averaged Navier Stokes equations (RANS) computational data. Various types of sampling designs for the low-fidelity data were examined to study to what extent the low-fidelity data contributed to improving the surrogate model versus only using limited high-fidelity data. Q. Zhang [27] applied the cokriging based data fusion algorithm on CFD data and data from industrial semi-empirical methods, like the US Air Force DATCOM (data compendium) to construct an aerodynamic characteristics database.

From the above analysis, two data fusion methods are commonly used in the domain of aerodynamics, that is, variable complexity modeling and cokriging. The target of the two methods is to generate a data set that is more accurate than low-fidelity data and greater in quantity than high-fidelity data. The implementation of the methods rely on the assumption that information of low-fidelity data are used to predict global trends while high-fidelity data are used to provide absolute value information and correct the global trends. The main ideas of the two methods are briefly explained below.

### 2.1. VCM

The two VCM approaches used in previous works were scaled approximations [28,29] and increment approximations. The scaling function σ(x) defines the ratio between a high-fidelity ($f_{h}$) and a low-fidelity ($f_{l}$) solution [30].

(1) $$\sigma \left(x_{s}\right)=\frac{f_{h}\left(x_{s}\right)}{f_{l}\left(x_{s}\right)},$$

where $x_{s}$ is the input variable at the observation points. The values of this scaling function σ(x) are interpolated throughout the whole design space. Then the function was approximated using the expression:

(2) $$f\left(x\right)=\sigma \left(x\right)f_{l}\left(x\right).$$

However, there are some potential problems associated with the scaling function approach when it combines low-and high-fidelity data. If the value of the low-fidelity data is exactly zero, the approach would not work. If the low-fidelity data are close to zero, σ(x) may be quite large and may amplify any approximation errors. To avoid these possible problems, the increment function β(x) was proposed instead of computing the ratio between the high- and low-quality data.

(3) $$\beta \left(x_{s}\right)=f_{h}\left(x_{s}\right)-f_{l}\left(x_{s}\right).$$

Similarly, the function is approximated using the equation:

(4) $$f\left(x\right)=\beta \left(x\right)+f_{l}\left(x\right).$$

This increment function is more reliable than a scaling ratio as the subtraction of small values does not result in any amplification errors. Kriging methods are commonly used to construct the increment surrogate model. The kriging surrogate model goes through the data points exactly, and can approximate the true model with fewer data. The fusion results can be guaranteed to be equal to the high-fidelity data completely, and the quality of the model is valid with small sample data sets.

### 2.2. Cokriging

To discuss the cokriging method, we first assume that low- and high-fidelity data sets are given as:

(5) $$X=\left[\begin{array}{c} X_{L}\\ X_{H} \end{array}\right]=\left[\begin{array}{c} X_{L}^{\left(1\right)}\\ \vdots \\ X_{L}^{\left(n_{L}\right)}\\ X_{H}^{\left(1\right)}\\ \vdots \\ X_{H}^{\left(n_{H}\right)} \end{array}\right],Y==\left[\begin{array}{c} Y_{L}\left(X_{L}\right)\\ Y_{H}\left(X_{H}\right) \end{array}\right]=\left[\begin{array}{c} Y_{L}\left(X_{L}^{\left(1\right)}\right)\\ \vdots \\ Y_{L}\left(X_{L}^{\left(n_{L}\right)}\right)\\ Y_{H}\left(X_{H}^{\left(1\right)}\right)\\ \vdots \\ Y_{H}\left(X_{H}^{\left(n_{H}\right)}\right), \end{array}\right]$$

where *X* is the sample point, *Y* is the response, and we assume $Y_{L}$ and $Y_{H}$ are two static stochastic processes:

(6) $$\left\{\begin{array}{c} Y_{L}=\mu_{L}+Z_{L}\left(x\right)\\ Y_{H}=\mu_{H}+Z_{H}\left(x\right) \end{array}.\right.$$

*Z* is the Gaussian correlation process, and we assume that the relation between $Z_{L}$ and $Z_{H}$ is:

(7) $$Z_{H}\left(x\right)=\rho Z_{L}\left(x\right)+Z_{d}\left(x\right),$$

where ρ is the scaling factor, and $Z_{d}$ represents the difference between $Z_{H}$ and $\rho Z_{L}$. Then, a covariance matrix can be constructed as follows:

(8) $$C=\left[\begin{array}{cc} cov(Y_{L},Y_{L}) & cov(Y_{H},Y_{L})\\ cov(Y_{L},Y_{H}) & cov(Y_{H},Y_{H}) \end{array}\right]=\left[\begin{array}{cc} \sigma_{L}^{2}\Psi \left(X_{L},X_{L}\right) & \rho \sigma_{L}^{2}\Psi \left(X_{H},X_{L}\right)\\ \rho \sigma_{L}^{2}\Psi \left(X_{L},X_{H}\right) & \rho^{2}\sigma_{L}^{2}\Psi \left(X_{H},X_{H}\right)+\sigma_{d}^{2}\Psi \left(X_{H},X_{H}\right), \end{array}\right]$$

where Ψ is the correlations between sample data points:

(9) $$\psi \left(X,X^{\prime }\right)=\prod_{k=1}^{n}e^{-\theta_{k}{\left|x_{k}^{i}-x_{k}^{j}\right|}^{p_{k}}}.$$

We can assume $\theta_{k}=\theta$ and $p_{k}=p$ based on the isotropic hypothesis [31]. The cokriging prediction model is given by:

(10) $$\hat{y}\left(x^{n_{e}+1}\right)=\hat{\mu }+cC^{-1}\left(y-I\hat{\mu }\right).$$

The meaning of $\hat{\mu }$, *c*, and other details of cokriging was discussed by A. I. J. Forrester [32]. The hyper-parameters in cokriging could be numerically estimated using a genetic algorithm or particle swarm optimization.

## 3. Methodology

### 3.1. Multilayer Perceptron

**Figure 1:**
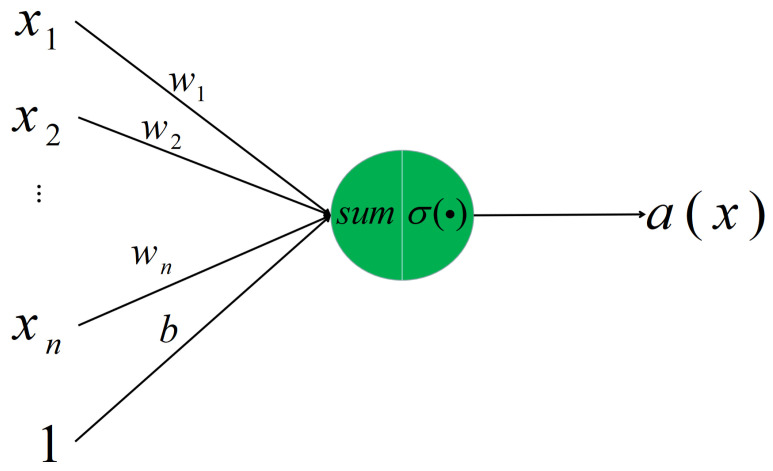
Typical neuron model.

The multilayer perceptron (MLP) [33] has the ability to extract the deep hidden features of information from data efficiently and accurately. The MLP is composed of several neurons, which are connected together in a complex manner to form a network [34]. Neurons are the basic elements of the MLP. Figure 1 is a typical neuron model: n+1 input, one output, and two computation functions.

The green circle represents a neuron that has n+1 inputs $x_{1},x_{2},\ldots ,x_{n},1$ and one output *a*, where $w_{1},w_{2},\ldots w_{n}$ are the corresponding weights to $x_{1},x_{2},\ldots ,x_{n}$ and *b* is a bias term. The arrow represents a weighted operation, through which the input $x_{i}$ will become $w_{i}x_{i}$. The neuron contains one summation function and one nonlinear activation function. The summation of the weighted inputs is

(11) $$\sum_{i=1}^{n}w_{i}x_{i}+b.$$

The weighted input is also called a weighted signal to the neuron. The output is obtained by passing the weight signal through a nonlinear activation function σ(g). Therefore, the output of the neuron is written as follows:

(12) $$a=\sigma (\sum_{i=1}^{n}w_{i}x_{i}+b).$$

**Figure 2:**
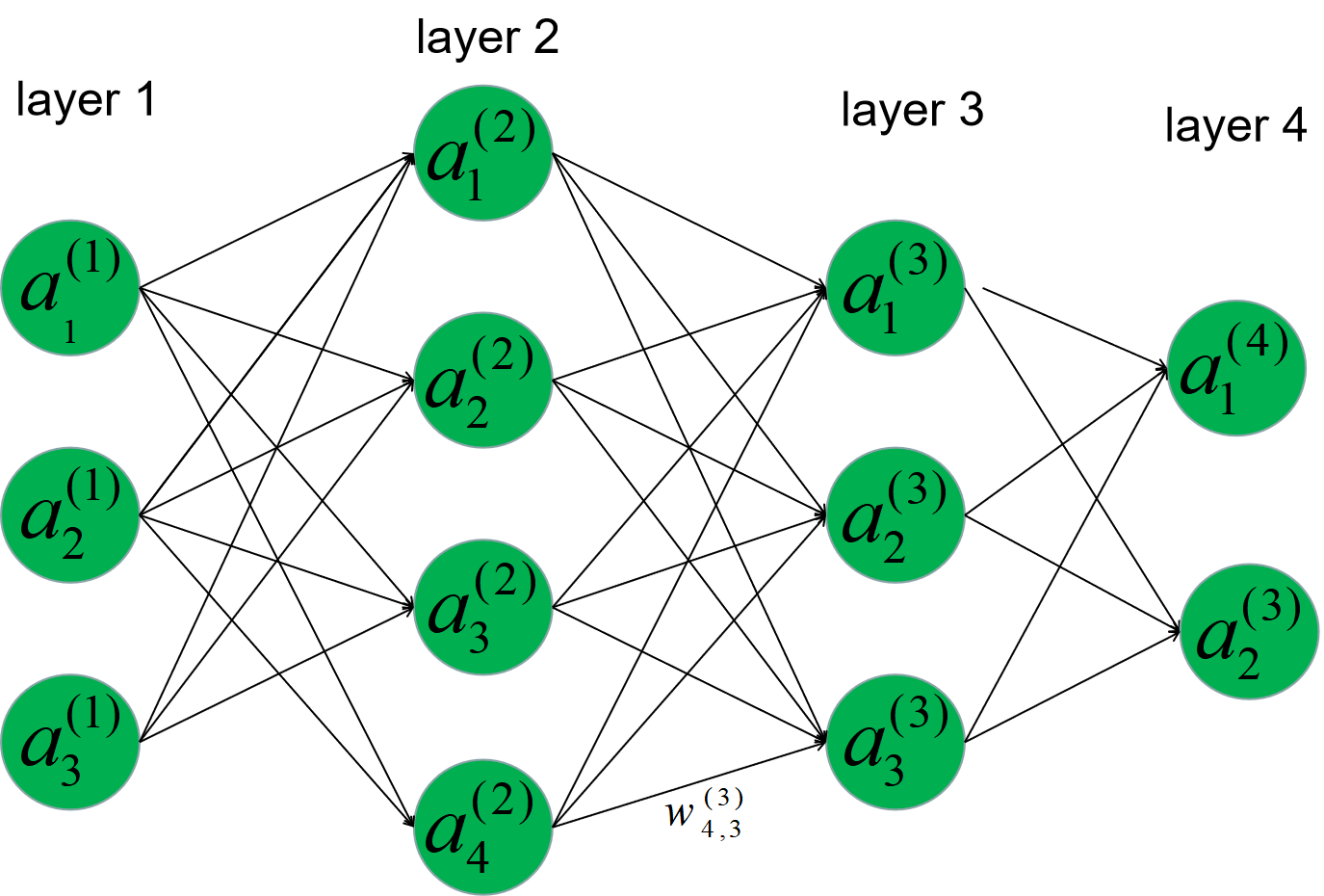
Typical multilayer perceptron (MLP) neural network.

Figure 2 is a typical MLP neural network, which consists of four layers with full connection. Layer 1 is called the input layer, layer 2 and layer 3 are called hidden layers, and layer 4 is called the output layer. The input layer receives the input signal from the user, and the other layers receive input signals from the previous layers. The output neuron is obtained as follows:

(13) $$a_{j}^{\left(k\right)}=\sigma \left(\sum_{i=1}^{n}w_{ji}^{k}x_{i}^{\left(k-1\right)}+b_{j}^{k}\right),$$

where $a_{j}^{\left(k\right)}$ represents the output of the *j*th neuron in the *k*th layer, $w_{ji}^{k}$ denotes the weight of the connection from the *i*th neuron in the (k−1)th layer to the *j*th neuron in the *k*th layer, $b_{j}^{k}$ indicates the bias term of the *j*th neuron in the *k*th layer, and σ(·) is the activation function. The process of obtaining the neuron activation is called feed forward. The weights and biases of the MLP are trained using a back propagation (BP) algorithm [35].

### 3.2. Multi-Fidelity Aerodynamic Data Fusion with Deep Neural Networks

The following descriptions of multi-fidelity deep neural networks are inspired by the works of Meng [17] and Babaee [36]. We assume that low-fidelity and high-fidelity data sets are given as $\left(X,Y_{L}\right)$ and $\left(X,Y_{H}\right)$. The correlation between low-fidelity and high-fidelity data can be expressed as:

(14) $$Y_{H}=f\left(X,Y_{L}\right),$$

where f(·) is a function that maps the data from the low-fidelity level to the high-fidelity level. Generally, there exist linear and nonlinear correlations between low-fidelity and high-fidelity data [16]; then, the function f(·) can be decomposed into the linear and nonlinear parts, which are expressed as

(15) $$f=f_{l}+f_{nl},$$

where $f_{l}$ and $f_{nl}$ denote the linear and nonlinear terms in *f*, respectively. To describe the contribution degree of the linear and nonlinear parts to the correlation between low-fidelity and high-fidelity data, a scaling hyper-parameter ρ is used. We can further write Equation (14) as

(16) $$Y_{H}=\rho f_{l}\left(X,Y_{L}\right)+\left(1-\rho \right)f_{nl}\left(X,Y_{L}\right),\rho \in \left[0,1\right].$$

The value of ρ is auto determined by training data. Now, we have constructed the correlation equation.

**Figure 3:**
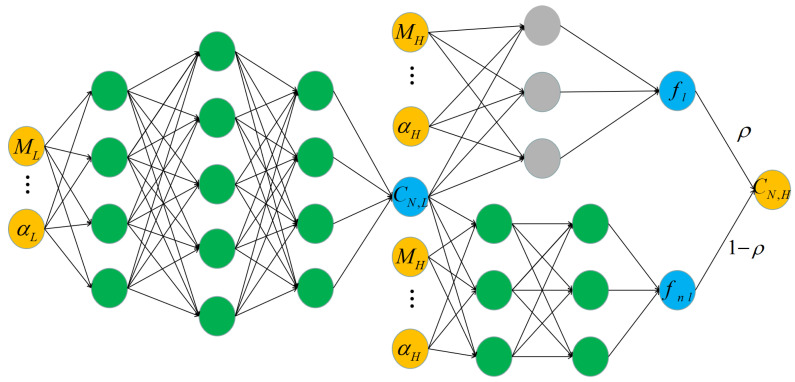
Architecture of the multi-fidelity aerodynamic data fusion model.

As shown in Figure 3, the proposed architecture of the multi-fidelity aerodynamic data fusion model based on deep neural networks is composed of three fully-connected neural networks with user flow conditions as inputs, for example, the Mach number, angle of attack α, sideslip angle β, and Reynolds number Re; and aerodynamic coefficients as outputs, for example, the coefficients of lift $C_{L}$, drag $C_{D}$, pitching moment $C_{M}$, normal force $C_{N}$, and axial force $C_{A}$. The three fully-connected neural networks can be employed to approximate the low-fidelity data, the linear part and nonlinear part of correlation for the low- and high-fidelity data. The hyperbolic tangent activation function is employed in the green neural network, and no activation function is included in the gray neural network due to the fact that it is used to approximate the linear part of the correlation. As to the number of fully connected layers, this depends on the complexity of the problem to be solved. $C_{N,L}$ and $C_{N,H}$ can be replaced by other coefficients.

The implementation, training, and predictions of the model were performed with the open-source software Pytorch. Machine learning techniques are broadly classified into supervised and unsupervised techniques. The work here is limited to supervised machine learning techniques, in which the neural network is trained using flow conditions as input data and aerodynamic coefficients as labels. Neural network training is an optimization process in which the unknown parameters are learned by minimizing the loss function. The commonly used loss functions include the mean square error (MSE), cross entropy, categorical hinge, and so forth. In the present study, the loss function for training samples is defined as follows:

(17) $$MSE=\frac{1}{N_{L}}\sum_{i=1}^{N_{L}}|y_{L}^{t}-y_{L}{|}^{2}+\frac{1}{N_{H}}\sum_{i=1}^{N_{H}}{\left|y_{H}^{t}-y_{H}\right|}^{2}+\lambda \sum w^{2},$$

where $y^{t}$ denotes the true value of the aerodynamic coefficients (labels). *y* is the output value of the networks, and *w* is weight of networks. $N_{L}$ and $N_{H}$ represent the number of low- and high-fidelity data sets, respectively. λ is the L2 regularization rate.

**Figure 4:**
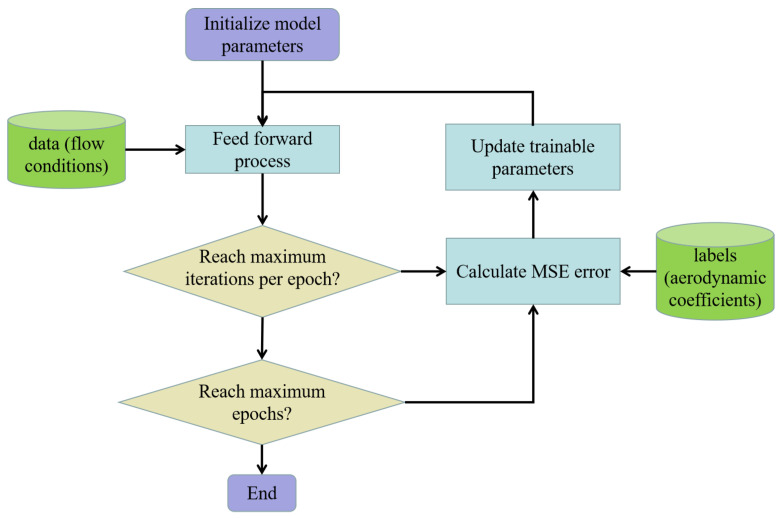
The training process of the proposed model.

The training process of the model is shown in Figure 4. The main training processes of the model are feed forward calculation and error back propagation. Forward calculation obtains prediction values through the output layer. Error back propagation transfers MSE errors backward through the algorithms, such as the gradient descent, and updated trainable parameters of the network, using an optimizer. This indicates a single training iteration with a batch, which is a subset of the training set. An epoch is the full pass of the training process over the entire training set. After feed forward calculation and error back propagation, if the desired convergence was not achieved, the above steps were repeated. The end of training can be determined by the error threshold or the maximal number of epochs. The latter is used as the end condition in this paper. In this paper, the trainable parameters were initialized with Xavier’s initialization method. The loss function was optimized using a stochastic gradient descent algorithm called Adam [37]. Adam is a first-order gradient-based algorithm that uses an adaptive learning rate based on past gradient information for each parameter update. So far, Adam has proved to be a good choice of algorithm for deep learning [38].

## 4. Results and Discussion

We studied a two-dimensional case to verify the proposed aerodynamic data fusion method. As a comparison, VCM and the cokriging method were used to predict the aerodynamic coefficients. The VCM in this case was an increment approximation that used the kriging model to approximate both low-fidelity data and increment data, and we called this the VCM-kriging method in the following.

### 4.1. Data Preparation

**Figure 5:**
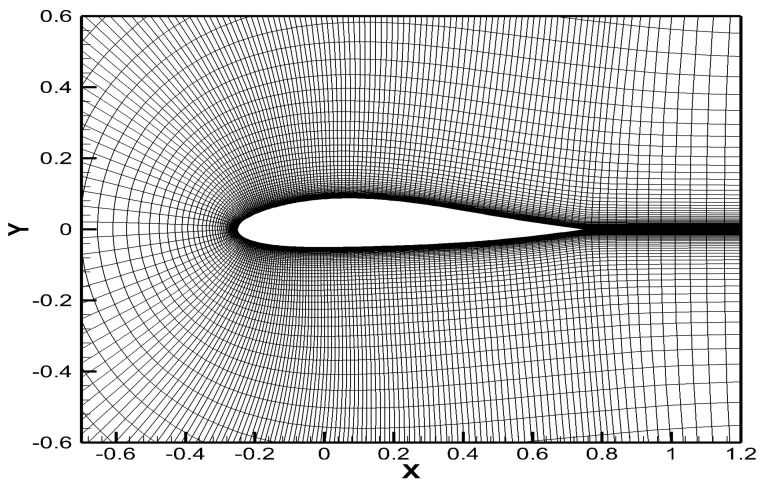
The tested airfoil sharp and computational grid.

Data are the carriers of information and data preparation is an important task in machine learning. As a test of the proposed aerodynamic data fusion approach, low-fidelity (Euler) and high-fidelity (Navier–Stokes) simulations were calculated for an airfoil sharp at various Mach numbers and angles of attack to obtain the aerodynamic force data. The tested airfoil shape, which is a variant of symmetric airfoil NACA0012, is shown in Figure 5. It was generated by improved Hicks-Henne bump function [39]. In this study, the number of bump functions was set to 8, and the values of the 8 control points were set to 0.0, 0.005, 0.0, 0.0, 0.005, 0.01, 0.005 and 0.01 respectively.

The aerodynamic coefficients of the airfoil were calculated using the computational fluid software MBNS2D [40], which was independently developed by our department. A Navier–Stokes (NS) equation, Roe scheme, and a two-equation k-ω SST (Shear Stress Transfer) turbulence model were adopted for this simulation. The Reynolds number took a fixed value of $6.5\times 10^{6}$. Figure 5 also shows the X–Y plane of the computational grid. The number of grids was set to 300×100, the grids of the leading and trailing edges were encrypted, and the first layer height in the wall-normal direction was less than $10^{-5}$ C (C is the chord length). It took approximately 350 seconds to calculate one aerodynamic coefficient at certain flow conditions with a personal computer (Intel Core i5-8250U CPU, 8G memory, and GeForce MX150 graphics card).

To study the gird convergence, four grid levels were chosen, and the grid refinement ratio was $\sqrt{2}$. Table 1 shows the simulation results of $C_{D}$ and $C_{L}$ at a fixed-flow condition. The relative error of Grid 2 was 3.42% and 0.8%. The convergence ratios $R_{G}$ of Grids 1, 2, and 3 were 0.42 and 0.53. 0 < $R_{G}$ < 1 indicates that the simulations were monotonically convergent [41].

In this case, we considered a two-dimensional aerodynamic force data fusion problem. To illustrate the problem, the case presented here includes the Mach number *M* and angle of attack α as input variables, and the longitudinal coefficients lift $C_{L}$ and drag $C_{D}$ were modeled. The data obtained by Euler simulation was used as the low-fidelity level and the data obtained by the Navier–Stokes simulation was used as the high-fidelity level. There were 120 and 24 training points at the low- and high-fidelity levels, respectively. The training data encompassed α and *M* values in the ranges:

(18) $$\left\{\begin{array}{c} M_{L}^{train}=\left\{-0.1,0.2,0.3,0.4,0.5,0.6\right\}\\ \alpha_{L}^{train}=\left\{-4^{o},-3^{o},-2^{o},-1^{o},0^{o},1^{o},2^{o},3^{o},4^{o},5^{o},6^{o},7^{o},8^{o},9^{o},10^{o},11^{o},12^{o},13^{o},14^{o},15^{o}\right\}\\ M_{H}^{train}=\left\{-0.1,0.4,0.6\right\}\\ \alpha_{H}^{train}=\left\{-4^{o},0^{o},1^{o},5^{o},7^{o},9^{o},11^{o},13^{o},15^{o}\right\}. \end{array}\right.$$

**Figure 6:**
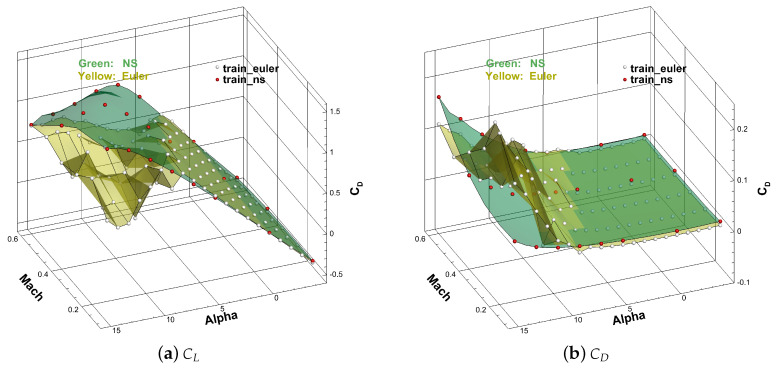
The low- and high-fidelity solution surfaces and training points: (**a**) the lift coefficient; (**b**) drag coefficient.

As illustrated in Figure 6, the green and yellow surfaces represent the high-fidelity solution (Navier–Stokes simulation) and low-fidelity solution (Euler simulation) of $C_{L}$ and $C_{D}$, respectively, and the white and red points are the training data obtained from the green and yellow surfaces.

### 4.2. Model Training

**Figure 7:**
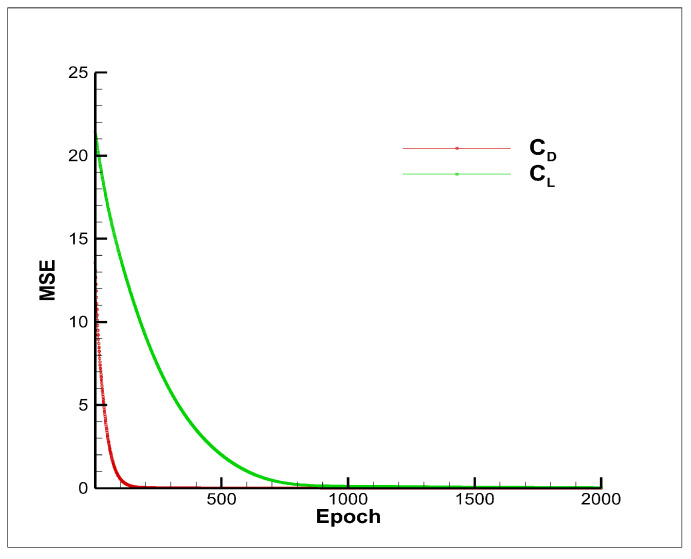
The training history.

In this case, there were four hidden layers used within the low-fidelity neural network, while two and one hidden layers were used within the nonlinear and linear neural networks, respectively. The training parameters of the proposed model were as follows: the maximal number of epochs was set to 80,000, the regularization rate was set to $\lambda =2.5\times 10^{-5}$ with a learning rate of 0.0001. The training convergence of the MSE for DNN architectures is shown in Figure 7, where the green and red lines indicate the convergence process of $C_{L}$ and $C_{D}$. For better observation, only MSE of the first 2000 epochs are drawn. The training MSE of $C_{L}$ and $C_{D}$ already reached steady-state values at around 1000 epochs and 200 epochs, before which the weights of the network were rapidly tuned to optimize the prediction model. As illustrated in Table 2, it cost approximately 204 and 201 seconds to train the prediction model of $C_{L}$ and $C_{D}$ with the same personal computer. The training time of the model was closely related to the number of training data and the number of epochs. The VCM-kriging and cokriging methods cost less time to train.

### 4.3. Discussion

In order to test the performance of the prediction model constructed before, a validation data set was generated with the Navier–Stokes simulation. There were 96 test points that encompass the Mach number and the angle of attack values in the ranges:

(19) $$\left\{\begin{array}{c} \left\{\begin{array}{c} M^{test}=\left\{0.2,0.3,0.5\right\}\\ \alpha^{test}=\left\{-4^{o},-3^{o},-2^{o},-1^{o},0^{o},1^{o},2^{o},3^{o},4^{o},5^{o},6^{o},7^{o},8^{o},9^{o},10^{o},11^{o},12^{o},13^{o},14^{o},15^{o}\right\} \end{array}\right.\\ \left\{\begin{array}{c} M^{test}=\left\{-0.1,0.4,0.6\right\}\\ \alpha^{test}=\left\{-3^{o},-2^{o},-1^{o},1^{o},,2^{o},3^{o},4^{o},6^{o},8^{o},10^{o},12^{o},14^{o}\right\}. \end{array}\right. \end{array}\right.$$

As illustrated in Table 2, the prediction time of 96 test points with all three methods was less than 1 s, which was very short and could be ignored; therefore, the time consumption of this method was mainly during the model training. The training time of the three surrogate models was shorter than that of one CFD evaluation cost, which was approximately 6 min (350 s). CFD often needs to calculate hundreds of flight states in practical applications; thereby, the CFD method consumes more time.

**Figure 8:**
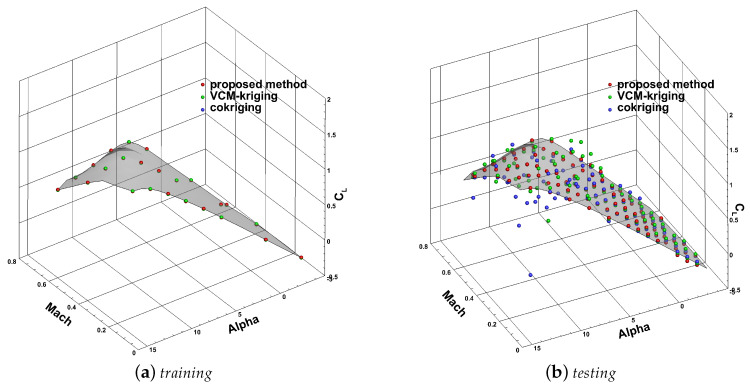
The predicted results of $C_{L}$ with different methods: (**a**) training; (**b**) testing.

**Figure 9:**
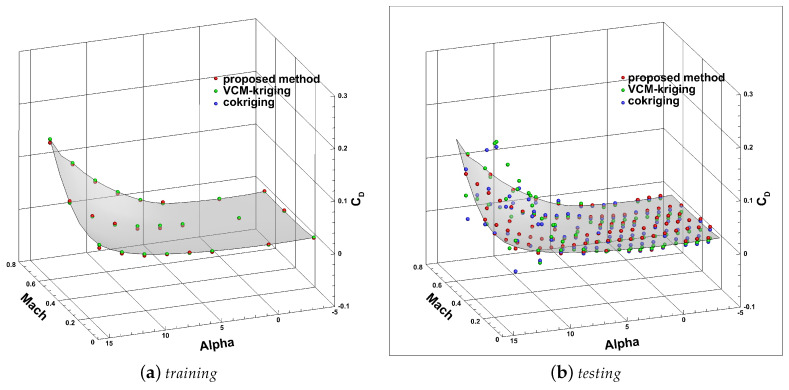
The predicted results of $C_{D}$ with different methods: (**a**) training; (**b**) testing.

Figure 8 and Figure 9 show the predicted results of the lift and drag coefficients at training points of high-fidelity data and test points, respectively. The colored surface in the diagram represents the true high-fidelity values. The red, green, and blue points represent the predicted values with the proposed method, VCM-kriging, and cokriging, respectively. Clearly, the predicted values of the training points by all three methods almost coincide with the expected surface. As to the test points, the predicted results of the proposed method were satisfactory, and the results of other two methods were not. Thus, we can draw an intuitive conclusion that the model established in this paper could accurately predict the two aerodynamic coefficients.

**Figure 10:**
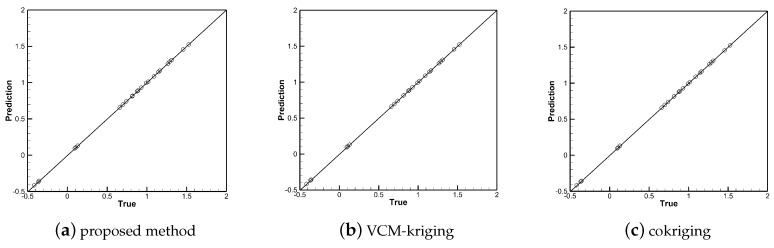
Accuracy spread (true vs. prediction) of the high-fidelity training points of $C_{L}$ with different methods.

**Figure 11:**
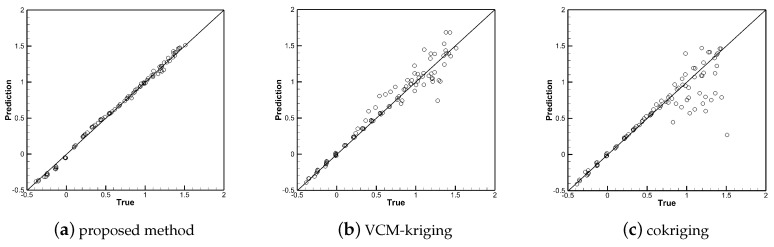
Accuracy spread (true vs. prediction) of the testing points of $C_{L}$ with different methods.

**Figure 12:**
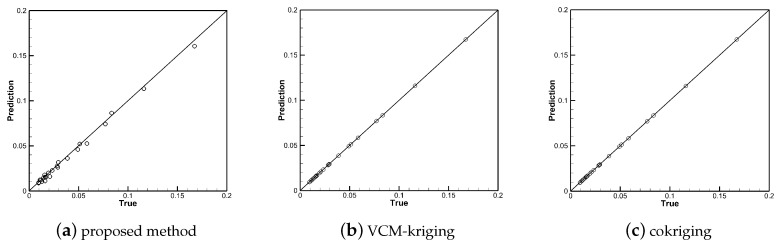
Accuracy spread (true vs. prediction) of the high-fidelity training points of $C_{D}$ with different methods.

**Figure 13:**
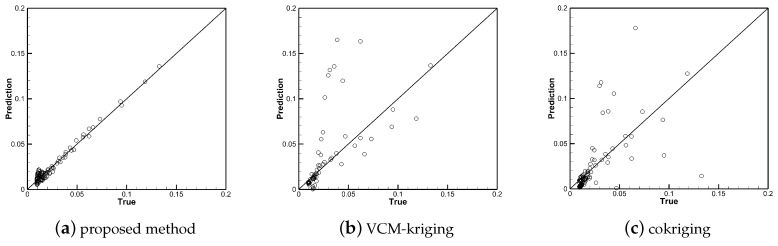
Accuracy spread (true vs. prediction) of the testing points of $C_{D}$ with different methods.

Figure 10, Figure 11 and Figure 12 show the linear regressions of true and predicted aerodynamic coefficients. The qualitative accuracy spreads of the training samples of the three methods are shown in Figure 10 and Figure 12. All points are clustered along the 45 deg line, the predicted values are close to the true value, which shows that all three methods show good performance in the training data sets, and the points fall accurately on the 45 deg line in subfigures (b) and (c). The qualitative accuracy spreads of the testing samples are shown in Figure 11 and Figure 13, the points are clustered along the 45 deg line in subfigure (a), while many points are located far away the 45 deg line in subfigures (b) and (c). Thus, the performance of the proposed method is better than that of VCM-kriging and cokriging in the testing data set for the test problem.

In addition, for further performance demonstration purposes, the root mean square error (RMSE) and average relative deviation error (ξ) were used to describe the accuracy of the results. The definitions are as follows:

(20) $$RMSE=\sqrt{\frac{1}{N}\sum_{i=1}^{N}{\left(y_{t}^{i}-y_{p}^{i}\right)}^{2}}$$

(21) $$\xi =\frac{\sum_{i=1}^{N}\left(y_{t}^{i}-y_{p}^{i}\right)}{\sum_{i=1}^{N}\left(y_{t}^{i}\right)},$$

where *N* represents the total number of training or testing samples, $y_{t}$ is the true value (high-fidelity value), and $y_{p}$ is the predicted value. The errors of the training and testing data with different methods are listed in Table 3. The proposed method has a small root mean square error and average relative deviation error for the training and testing prediction, which indicates that the proposed method in this paper was able to perform well for both $C_{L}$ and $C_{D}$ prediction. The VCM-kriging and cokriging method demonstrated perfect performance for the training set prediction, where both the root mean square error and average relative deviation error were 0. This is because the kriging and cokriging surrogate models go through the training data points exactly. However, the VCM-kriging and cokriging method were not able to perform as well as the proposed method for both $C_{L}$ and $C_{D}$ prediction on the testing set, where the average relative deviation error value was 58.40% and 82.98% for $C_{D}$ prediction compared with 12.89% of the proposed method.

## 5. Conclusions

Data fusion technology offers great potential and prospects in the field of aerodynamics analysis. In this paper, we applied a deep neural network algorithm to fusion information contained in the multi-fidelity aerodynamic data, which provides an effective way to generate more high-quality aerodynamic data with less cost. The proposed method can learn the linear and nonlinear correlations between the low- and high-fidelity using the training sample data. The structure, training process, optimizer, and so forth of the proposed model were introduced. In the study case, both the root mean square error and average relative deviation showed that the proposed method had better performance for the test problem compared with the VCM-kriging and cokriging methods.

Although, the case only studied the multi-fidelity data obtained by different CFD solutions (Euler and Navier–Stokes), the method can also be extended to wind tunnel or flight test data. Compared with the traditional VCM-kriging and cokriging methods, the deep neural network-based fusion method had obvious advantages in high-dimensional or large-scale data problems. Therefore, more input variables can be dealt with using the method for complex aerodynamic problems involving more influence factors, and more data points can be used to build the fusion model. Thus, a large amount of historical data can be fully utilized. Despite the advantages above, an iterative process is used to determine the optimal hyper-parameters of the network, and this is tedious work for even experienced engineers to adjust the hyper-parameters (e.g., the regularization rate) in dealing with new tasks. Future work may include experimental design of the training points by considering the historical information, expert experience, or wing geometry features. Overall, the deep neural network based multi-fidelity aerodynamic data fusion method is a promising method and can be widely applied across the spectrum of engineering.

## Figures and Tables

**Table 1:** The computational-fluid-dynamics (CFD) results of $C_{D}$ and $C_{L}$ (α = 2, Ma = 0.4, Re = $6.5\times 10^{6}$).

Grid	$C_{D}$	ϵ (%)	$R_{G}$	$C_{L}$	ϵ (%)	$R_{G}$
gird1 (210×70)	0.01088	9.46	0.42	0.34306	1.2	0.53
gird1 (300×100)	0.01028	3.42		0.34443	0.8	
gird1 (420×140)	0.01003	0.9		0.34516	0.6	
gird1 (600×200)	0.00994	-	-	0.34740	-	-

**Table 2:** The time consumption of different methods.

Time	Coefficient	Proposed Method	Variable Complexity Modeling (VCM)-Kriging	Cokriging
Training time	$C_{L}$	204 s	53 s	61 s
	$C_{D}$	201 s	50 s	60 s
Prediction time	$C_{L}$	<1 s	<1 s	<1 s
	$C_{D}$	<1 s	<1 s	<1 s

**Table 3:** Errors of the training and testing data with different methods.

Set	Method	Root Mean Square Error (RMSE)		ξ	
		$C_{L}$	$C_{D}$	$C_{L}$	$C_{D}$
train	proposed method	0.0024	0.0029	0.0023	0.0584
	VCM-kriging	0.0	0.0	0.0	0.0
	cokriging	0.0	0.0	0.0	0.0
test	proposed method	0.0333	0.0038	0.0389	0.1289
	VCM-kriging	0.1220	0.0382	0.1110	0.8298
	cokriging	0.2240	0.0296	0.1627	0.5840
